# Air ambulance services in the Arctic 1999-2009: a Norwegian study

**DOI:** 10.1186/1865-1380-1-1

**Published:** 2011-01-27

**Authors:** Jan Norum, Trond M Elsbak

**Affiliations:** 1 Department of Clinical Medicine, Faculty of Health Sciences, University of Tromsø, N-9037 Tromsø, Norway; 2 Department of Oncology, University Hospital of North Norway, N-9038 Tromsø, Norway; 3 Northern Norway Regional Health Authority, N-8038 Bodø, Norway

## Abstract

**Background:**

Air ambulance services in the Arctic have to deal with remote locations, long distances, rough weather conditions and seasonable darkness. Despite these challenges, the people living in the area expect a high quality of specialist health care.

**Aims:**

The objective of this study was to analyse the air ambulance operations performed in the Norwegian Arctic and study variations in diagnoses and flight patterns around the year.

**Methods:**

A retrospective analysis. All air ambulance operations performed during the time 1999 - 2009 period were analysed. The subjects were patients transported and flights performed. The primary outcome measures were patients' diagnoses and task patterns around the year.

**Results:**

A total of 345 patients were transported and 321 flights performed. Coronary heart and vascular disease, bone fractures and infections were the most common diagnoses. Most patients (85%) had NACA score 3 or 4. Half of all fractures occurred in April and August. Most patients were males (66%), and one fourth was not Norwegian. The median flying time (one way) was 3 h 33 m. Ten percent of the flights were delayed, and only 14% were performed between midnight and 8.00 AM. The period April to August was the busiest one (58% of operations).

**Conclusions:**

Norway has run a safe air ambulance service in the Arctic for the last 11 years. In the future more shipping and polar adventure operations may influence the need for air ambulances, especially during summer and autumn.

## Introduction

The Northern Norway Regional Health Authority (NNRHA) trust is responsible for the specialist health care service and all patient transportations in northern Norway. This includes the Norwegian Arctic areas (Svalbard, Bear Island, Hopen and Jan Mayen). Svalbard is a group of islands reaching up to the 80th degree northern latitude and covers an area of 61,020 km^2^. The land area constitutes 16% of Norway. The largest island is Spitsbergen, and its municipalities are Lonyearbyen, Barentsburg, Svea, Hornsund and Ny-Ålesund. The main airport is located at Longyearbyen, but there is also a minor one at Svea. The main industries on the island are coal mines, tourism, education, research and satellite services. According to data from Statistics Norway http://www.ssb.no, as of January 2009 there were a total of 2,570 inhabitants (2,085 Norwegians, 470 Russians and 10 Poles) on the island. There is a rich fishing area in the Svalbard zone, and fishermen from various nations such as Great Britain, Germany, Spain, Portugal, Russia, Iceland, the Faroe Islands and Norway fish in the area.

Whereas people living in the Arctic experience seasonable darkness and polar nights, the summer is light. For example, Longyearbyen experiences 4 months of seasonable darkness (no sun; 7 October - 8 March), including 2 months (14 November - 30 January) with complete darkness ("polar night"). "In compensation", there are 4 months of midnight sun during the summer (20 April - 23 August).

The Norwegian health care service in the region is provided by a small hospital unit in Longyearbyen. The unit is run by the University Hospital of North Norway (UNN) trust and is staffed with three medical doctors (one surgeon and two general practitioners). According to standard procedure, at least one doctor stays on the island at all times. Due to the limited staff, the hospital serves as a "preparedness hospital" taking care of primary health care, casualties and emergency care. Rough weather conditions, often presenting with strong winds, ice, cold temperatures and seasonable darkness, introduce challenges to health care in the Arctic. Long distances and almost no alternatives for landing make it necessary to be very cautious concerning safety before and during flights. Peoples' activities in the Arctic vary significantly around the year. The coal mines have reduced activity during the summer, fisheries experience limited access to the northern regions in winter because of enlarged polar ice coverage, cruise liners mainly operate in the area during summer, and polar adventure activities employing dog sleds or snowmobiles mainly take place during periods with daylight and snow (mainly spring and autumn). Based on this knowledge, we aimed to clarify the variations in patients' diagnoses and flights pattern around the year.

## Methods

**Figure 1:**
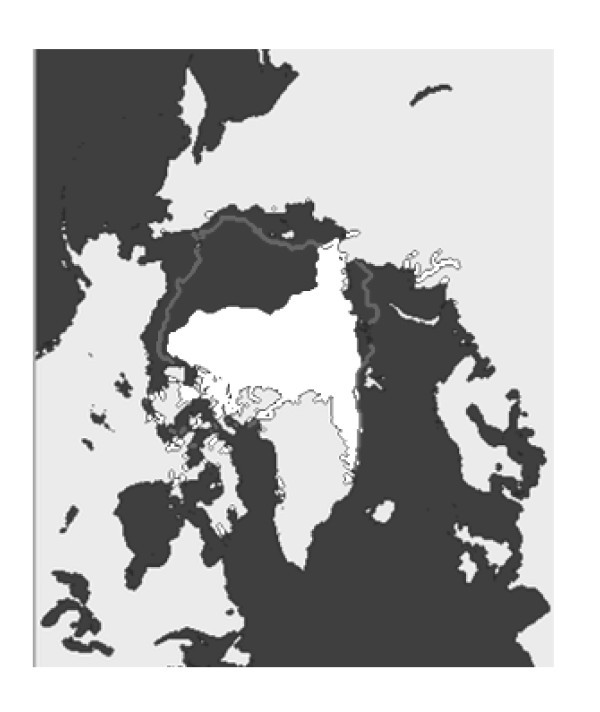
**The Arctic Sea **http://benmuse.typepad.com/ben_muse/arctic/. The *white cover *is the Arctic ice as in September 2007. The *arrows *points to Svalbard (*northern end*) and Tromsø (*southern end*).

The Arctic is shown in Figure 1. Despite the remote location, the population at Svalbard requests health care of similar quality to that offered on the mainland. An efficient air ambulance service is of utmost importance to meet these expectations. To administrate the service, the four Regional Health Authority (RHA) trusts have together established a company named Luftambulanse tjenesten ANS http://www.luftambulanse.no. The company registers all air ambulance activities in the LABAS database, employing a specific report sheet filled out by the medical crew (specialised nurse or medical doctor). Furthermore, they administrate finances for the supply of air ambulance operations. In northern Norway, the operations have been performed by the company Lufttrnsport AS http://www.lufttransport.no. They employ Beechcraft King Air 2002/B200 airplanes and Augusta AW 139 helicopters. Air operations by the Norwegian Coast Guard (NCG; employing Lynx helicopters onboard), the Governor of Svalbard (GoS; operating a Super Puma helicopter at Longyearbyen), the Norwegian Air Force (NAF; operating Sea King helicopters at Banak and Bodø on the Norwegian mainland) and other nationalities' prospective air operations in the region were not included. Due to range limitations, the Lynx and Super Puma helicopters in the region usually have to transport patients to Longyearbyen for treatment and/or transportation to the Norwegian mainland is carried out by airplane. Most patients taken care of by the Lynx or Super Puma crews are thus indirectly included in our survey. The NAF's Sea King helicopters operate on the Norwegian mainland and along its coastline. They very rarely operate in the Arctic.

In February 2010, the LABAS database was analysed focussing on the time period between 1 January 1999 and 31 December 2009. The primary goal was to clarify the variations in patients' diagnoses and flights pattern around the year. The following data were registered:

- Flight data: Date and time of start and end of task, time spent, state of emergency (non-urgent, urgent, emergent) according to the Norwegian Index for Medical Emergency Assistance [1], destination (hospital) and any delay of more than 15 min.

- Patient data: Sex, age, nationality, diagnosis (according to the international classification of diseases, ICD), oxygen support, intubation, analgesics given, degree of seriousness [National Advisory Committee on Aeronautics (NACA) scale; the scale is shown in Table 1], intravenous administrations and the use of vasopressor drugs.

**Table 1:** The National Advisory Committee on Aeronautics (NACA) scale.

Score level	Patient status
NACA 0	No injury or illness

NACA 1	Not acute life-threatening disease or injury

NACA 2	Acute intervention not necessary, further diagnostic studies needed

NACA 3	Severe, but not life-threatening disease or injury; acute intervention necessary

NACA 4	Development of vital (life-threatening) danger possible

NACA 5	Acute vital (life-threatening) danger

NACA 6	Acute cardiac or respiratory arrest

NACA 7	Death

### Statistical analysis

The Microsoft Office Excel 2007, Microsoft Corp., Redmond, WA, was employed for the calculations and database. Statistical Package for Social Science (SPSS) version 16.0, SPSS Inc., Chicago, IL, was employed for statistical analyses. Cases with an unknown value for a particular variable were excluded from analysis involving that variable. Statistical analyses were performed employing descriptive statistics and bivariate correlation analysis. All *P*-values are two tailed and considered statistically significant when *P *< 0.05.

No approval from the regional ethics committee was necessary as no individual patient identifiable data were accessed by the researchers.

## Results

**Figure 2:**
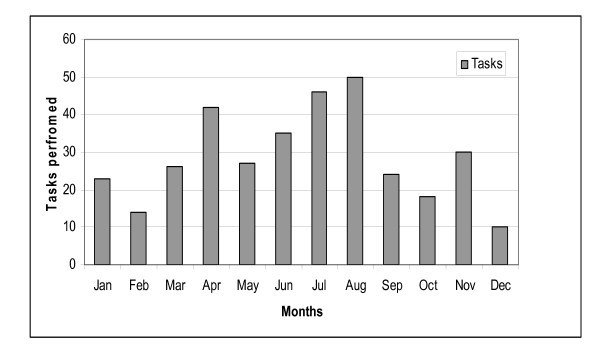
**The number of patients transported each month of the year**.

Three hundred forty-five patients and 321 flights were identified, and incidents were most common in April, June, July and August. Details are shown in Figure 2. Patient and task characteristics are shown in Table 2. Most patients (93%) were transported to the University Hospital of North Norway (UNN) (Figure 1).

**Table 2:** Overview of air ambulance operations in the Norwegian Arctic during the time period 1999 - 2009.

Items	Subgroup	Patients	%	Median age (yrs)	Range
***Patient characteristics***	All (n = 345)				
Age		345	100	47	0-92
Sex	Female	117	34	41	2-88
	Male	225	65	47	1-92
	Sex not registered	3	1		
***Nationality***	Not Norwegian (total)	75	22		
	Russian	31	9		
	German	8	2		
	Swedish	5	1		
	British	2	0.4		
	Finnish	1	0.2		
	Faroe Island	1	0.2		
	Unspecified	27	8		
***Task characteristics***					
Emergency status	Non-urgent	84	24		
	Urgent	144	42		
	Emergent	117	34		
Diagnosis	Psychiatry	9	3	42	21-67
	Infection	34	10	40	1-84
	Heart and vascular disease	76	22	54	19-87
	Bone fracture	71	21	48	2-80
	Gynaecology/obstetrics	28*	8	29	20-40
	Cancer/tumour	4	1	57	49-73
***Treatment***	Intubation	4	1		
	Oxygen	204	59		
	Analgesics	135	39		
	Vasopressors	34	10		

*Twenty-four of the patients were transported to give birth to their children on the mainland.

**Figure 3:**
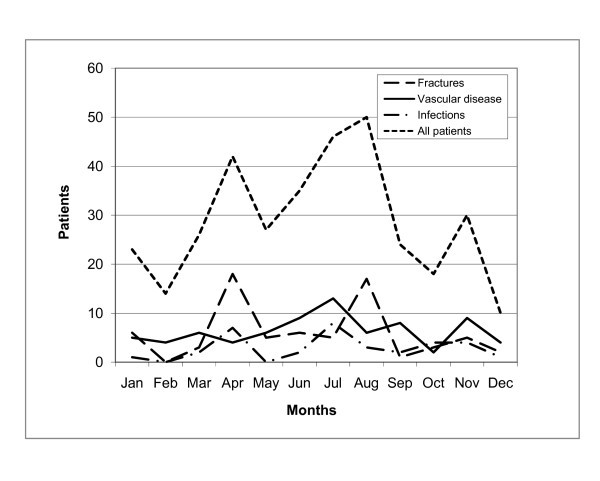
**The number of patients transported per month according to the main diagnostic groups (infection, vascular disease and fracture)**.

Heart and vascular disease together with bone fracture and infections were the most frequent diagnoses and constituted half of all cases (Table 2). One tenth of the patients had a gynaecologic/obstetric condition. Seasonal variations are shown in Figure 3. Half of all fractures occurred in April and August. The male/female ratio was 1.6 (inhabitants at Svalbard have a male/female ratio of 1.3). Fractures were more common among the age group 40 - 60 years, but there was no statistically significant correlation between age and fractures (*P *= 0.833).

**Figure 4:**
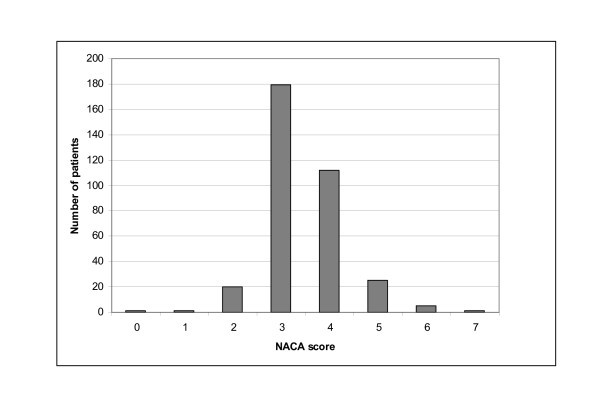
**The NACA score distribution among the patients**.

Concerning severity, most (85%) cases scored 3 or 4 on the NACA scale. Details are shown in Figure 4. There was a correlation between NACA score and age (*P *= 0.027). This was because heart and vascular disease was more common among the elderly. The mean NACA score among the heart and vascular disease group was 4.1 versus 3.3 among the controls. Three fourth of the cases were classified as urgent or emergent, and the state of emergency was correlated to heart and vascular disease (*P *= 0.020) and gynaecologic/obstetric causes (*P *= 0.000).

**Figure 5:**
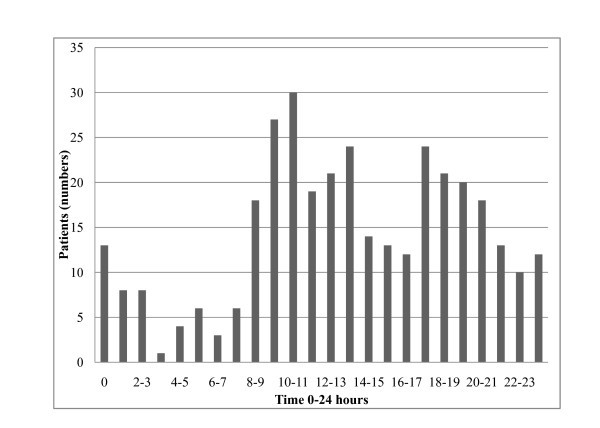
**The number of patients transported according to time (0 - 24 h) of takeoff (0 = midnight)**.

**Figure 6:**
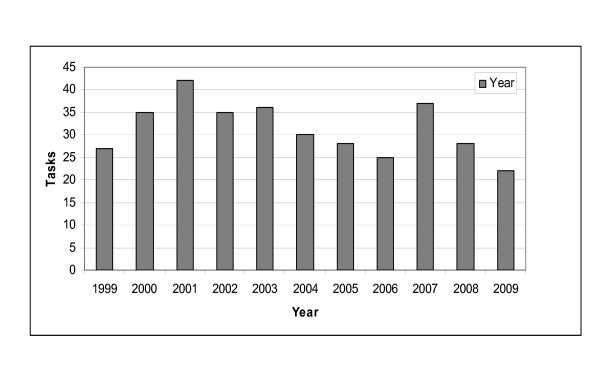
**The number of patients transported each year**.

Most patients were transported during the daytime. Only 50 patients were handled during periods of polar night and 97 (28%) during the period of seasonable darkness. Few (14%) patients were handled between midnight and 08:00 a.m. Details are shown in Figure 5. No increase in the number of tasks was revealed during the study period (Figure 6). The mean time spent per flight (one way) was 3 h 33 min (range 1 h - 8 h 35 min). Thirty-five transports were delayed, and the mean delay was 36 min. The specific cause of delay was not registered.

## Discussion

Heart and vascular disease together with bone fractures was the most frequent diagnosis. This has also been documented by other investigators [2,3]. Gynaecologic and obstetric causes accounted for 10% of patients. This is because pregnant women are routinely evacuated to the mainland for childbirth. A similar situation has been described among Canadian Inuit residents in the Canadian Arctic [4]. Researchers have commented that evacuation for childbirth has deleterious social and cultural effects [4]. Canadians have therefore recently established community birthing centres in Nunavik and Nunavut. This is not a current policy for the Norwegian Arctic because of the limited number of women living in the region, risk factors and the capabilities (no permanent access to a surgeon) at the local hospital unit.

The high frequency of fractures in August has also been documented in a prior Norwegian study from the Norwegian Coast Guard [5]. The authors argue that this situation is caused by the fact that workers and fishermen are less cautious when returning to work after their summer holiday.

In the future, stroke patients may become a growing group among the air ambulance patients because there will be more elderly people and new treatment guidelines. The role of air ambulance (helicopter) services in the transfer of stroke patients has been illustrated by Thomas and colleagues [6]. Prior to thrombolytic therapy, a CT scan has to confirm the diagnosis. Because of the limited time window between symptom onset and initiation of therapy, an air ambulance service is important for the hasty transfer of patients to the mainland for CT scanning.

We have documented the logistics of the air ambulance service in the Norwegian Arctic and the variations around the year. Whereas the geography of northern Norway makes it necessary to include both airplanes and helicopters in the service, many countries employ helicopter emergency medical services (HEMS) alone [7,8]. However, HEMS has limitations. In northern Norway rough weather conditions are a significant problem, especially during winter. A study from the region revealed an access rate of only 40% between November and March [9]. Even in northern Norway, ground transportation may be a good alternative [10,11]. However, because of the lack of roads and long distances, ground transportation is of limited value in the Arctic When appropriate, sea transport may be an alternative.

Despite the patients being airborne, there was a significant one-way flying time. Such a time span has also been shown employing heavy search and rescue helicopters in the Barents Sea [12]. The complexity of running operations in remote and cold regions has been illustrated in Antarctica [13]. In this report a ski-equipped airplane was employed, and a critically ill patient was transported 9 h north to New Zealand.

Daylight returns to Svalbard in early March, and in April people perform many outdoor activities. Dog sleds and especially snowmobiles are common means of transport. The sunlight reflected by the white cover of snow may cause "white out" and consequently an increased risk of accidents. Svalbard has the highest frequency of snowmobile accidents worldwide [14-16]. During a 3-year period (1997 - 2000), 107 snowmobile injuries were registered [15]. Most injuries (79.4%) occurred in the time period between March and May. This is in accordance with the peak of fractures in April shown in our survey.

In the future, significant changes will occur in the Arctic. As the ice is melting because of worldwide climate changes, shipping may take advantage of new routes among Europe, Northern Russia, Asia and North America. Furthermore, polar adventure operations will become steadily more popular as the coast areas of northern Greenland and north of Svalbard will be available for longer periods. In such a setting, Svalbard may become an important base for air ambulance services in the Arctic.

Air ambulance service is costly and limited in terms of resources, especially in the Arctic [17]. It should therefore be discussed whether passengers participating in polar adventure operations must have a declaration from their personal physician that they are fit for the journey. Such operations are very different from "tropical" cruise lines [18].

To achieve maximum value (health gain) for the money, an excellent fleet coordination system is mandatory. Furthermore, high-quality decision criteria for aeromedical evacuation are important [19-21]. This has been summarised with the words "right patient, place and time" [20]. Another limitation has been the access to competent crew members [22]. As long as the Norwegian government decides to keep and support the Norwegian municipalities on Svalbard, a basic health care infrastructure has to be funded. In light of the potential (shipping, fishery, oil/gas industry) development of the Arctic region, a parallel expansion of the health care infrastructure should be considered.

## Conclusion

The NNRHA trust has been responsible for safe air ambulance operations in the Arctic, serving both Norwegians and others. The pressure on the limited resources is strongest in April, June, July and August. In the future, shipping and polar adventure operations will increase the need for health care services in the Arctic, especially during summer and autumn. This should be focussed on in future model-based analysis.

## Author information

The author is a medical oncologist, professor at the Faculty of Medicine at the University of Tromsø and medical director at the North Norway Regional Health Authority.

## Competing interests

The authors declare that they have no competing interests.

## Authors' contributions

Both TME and JN took part in the design of the study. TME collected the data from the LABAS database and made overviews of the material. JN carried out the statistical analysis, searched the PubMed database for relevant studies/reports and wrote the article. All authors read and approved the final manuscript.
